# Four cases report: Treatment of knee joint cartilage defects using autologous chondrocyte patch implantation

**DOI:** 10.3389/fsurg.2022.1015091

**Published:** 2022-11-08

**Authors:** Le Wang, Han Li, Yiguo Cao, Cheng Song, Qi Chen, Jun Hao, Weiguo Zhang, Kang Tian

**Affiliations:** ^1^ Department of Joint and Sports Medicine, First Affiliated Hospital, Dalian Medical University, Dalian, China; ^2^ Department of Nuclear Medicine, First Affiliated Hospital, Dalian Medical University, Dalian, China

**Keywords:** cartilage defect, autologous chondrocyte patch implantation, sandwich technique, cartilage repair, ACI, MACI

## Abstract

**Introduction:**

Autologous chondrocyte implantation (ACI) is a crucial method for the treatment of defects in articular cartilage. However, the extant methods for the preparation of autologous chondrocyte patch are relatively complicated and money-consuming. Therefore, an efficient, reliable, easy-to-follow, and cost-effective technique is needed to overcome constraints. This case report aims to introduce an autologous chondrocyte patch fabrication technique to repair knee joint cartilage defects and report our typical cases with a 2-year follow-up.

**Case presentation:**

We described four cases in which patients complained of knee joint pain. According to radiological examination, the patients were diagnosed as knee joint cartilage defect. Arthroscopy and autologous chondrocyte patch implantation were performed as well as a 2-year follow up of patients. The autologous chondrocyte patch for knee joint cartilage repair was fabricated using a “sandwich” technique. The preoperative and postoperative knee function was evaluated by four subjective evaluation systems. MRI was performed for all patients to achieve more intuitionistic observation of the postoperative radiological changes of defect sites. The quality of repaired tissue was evaluated by Magnetic Resonance Observation of Cartilage Repair Tissue (MOCART). Postoperative follow-up showed improvement in clinical and MOCART scores for all patients. However, one patient complained of knee joint pain after walking for a long time or recreational activities from 12- to 18-month postoperatively. The location of pain for this patient was not in accordance with the location of cartilage defect.

**Conclusion:**

The patients undergoing autologous chondrocyte patch implantation demonstrated clinical improvement and good quality of repaired tissue postoperatively. The procedure is an efficient and cost-effective treatment for knee joint cartilage defect in this report. In addition, patients with osteoarthritis carry the risk of a poor outcome after the procedure, and whether to have a procedure should be considered carefully.

## Introduction

A defect in articular cartilage is a common orthopedic problem, especially in the weightbearing areas of lower-extremity joints (e.g., knee). It has a high prevalence of morbidity in the general population (5%) (1, 2). The lack of vascular, nervous, or lymphatic systems hinders articular cartilage from healing. It can trigger pain, swelling, and dysfunction in the joint (3).

Autologous chondrocyte implantation (ACI) is a crucial method for the treatment of defects in articular cartilage. It can be divided into three generations (4). The first generation (ACI-P) was reported first by Brittberg et al. in 1994, and has two steps. During the first step, arthroscopy is undertaken to obtain chondrocytes, and then they are sent for culture. The second step involves injecting a suspension of cultured chondrocytes below a periosteal patch after 14–21 days (5). The second generation (ACI-C) uses a bioabsorbable collagen membrane instead of a periosteal patch. The third generation (which is based on the second generation) involves the creation of cartilage-like tissue in a biodegradable scaffold and is named ACI-M or matrix-induced autologous chondrocyte implantation (MACI) (6, 7). Several studies have revealed that ACI can lead to effective short-, medium-, and long-term outcomes (4, 7–13). However, the extant methods for the preparation of autologous chondrocyte patch are relatively complicated and money-consuming. Therefore, an efficient, reliable, easy-to-follow, and cost-effective technique is needed to overcome constraints. Therefore, we introduce an autologous chondrocyte patch fabrication technique to repair knee joint cartilage defects and report our typical cases with a 2-year follow-up for clinical and radiological outcomes in this report.

## Case report

Case 1: A 26-year-old male sustained an injury to his left knee while fishing. He experienced a twisting of his knee, while a varus impaction force was applied to the slightly flexed knee. He visited the outpatient department of other hospital and was treated with conservative treatment. After 1 year, he had left knee sprain again, accompanied by severe knee joint pain and swelling. Case 2: A 36-year-old female suffered discontinuous pain in left knee that continued for at least 16 years. There was no history of obvious trauma. She had frequent knee joint pain and motion restriction in the past 1 month before hospitalisation. Case 3: A 47-year-old male fell while running 14 years ago. The right knee joint pain was worse with activity and decreased with rest. At that time, he was not receiving any examination or therapy. The right knee joint pain was aggravated in recent months before hospitalisation. Case 4: A 37-year-old male fell while running. After that, he suffered from persistent left joint pain. He could not walk up or down stairs and the quality of his life was seriously influenced.

All these four patients were diagnosed as knee joint cartilage defect after physical and MR examination during hospitalization. Demographic information (sex, age, height, weight), medical and previous history, and cartilage defect characteristics of these patients (length, width, depth, shape, localization) were documented (Supplementary Table S1).

Surgical treatments were performed for patients. The standard procedure involved two steps. The first step was knee arthroscopy in which healthy cartilage tissue was removed from the intercondylar fossa (non-weightbearing areas) and the characteristics of the cartilage defect (length, width, depth, shape, localization) were checked.

**Figure 1:**
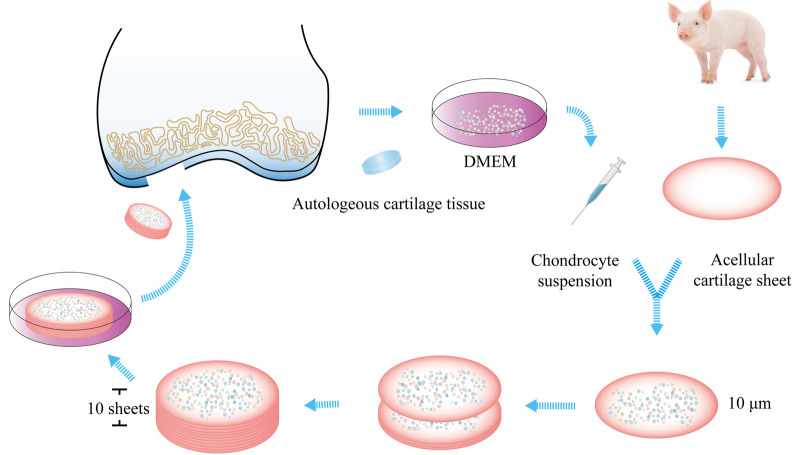
Fabrication of the patch. Healthy cartilage was harvested from the intercondylar fossa. Chondrocytes were isolated enzymatically. The cartilage used for the fabrication of acellular cartilage sheets was harvested from the ears of adult pigs. It was cut into sheets, and the sheets were decellularized. Chondrocytes were seeded on the sheet. Then, another acellular cartilage sheet was superposed on the first sheet, and these procedures were continued until ten sheets were stacked together. The construct was cultured at 37°C in an atmosphere of 5% CO_2_ and 95% relative humidity for 4 weeks before implantation.

The healthy removed cartilage tissue was stored in Dulbecco's modified Eagle's medium (DMEM, Absin, Shanghai, China) at 4°C and sent immediately to the laboratory for cell cultivation. Chondrocytes were isolated enzymatically after digestion by 1% collagenase II (Absin, Shanghai, China) at 37°C for 4 h. Then, they were expanded using culture conditions for autologous cells. DMEM was supplemented with 10% autologous serum (separated from peripheral blood), L-glutamine, and antibiotics (penicillin and streptomycin). Cells were cultured at 37°C in an atmosphere of 5% CO_2_ and 95% relative humidity. The medium was removed, and a fresh medium was added every three days. A maximum of two passages were undertaken for each culture. The cartilage used for fabrication of acellular cartilage sheets was harvested from the ears of adult pigs. First, the cartilage was cut into a cylindrical shape with a diameters of 2 cm. Then, the cylindrical cartilage was cut into sheets (using a freezing microtome) of thickness 10-μm. The sheets were decellularized in 1% sodium dodecyl sulfate (SDS, Coolaber, Beijing, China) for 24 h. After decellularization, the sheets were rinsed thrice in sterile water. A vacuum freeze-drier was used for lyophilization of the sheets. The diameter of sheets was narrowed to be about 1.8 cm. An acellular cartilage sheet was placed in a culture dish, and 5-μl of the chondrocyte suspension was seeded on it. The concentrations of chondrocyte suspension was 20 × 10^6^ cells/ml. Then, another acellular cartilage sheet was superposed on the first acellular cartilage sheet with 5-μl of the chondrocyte suspension seeded on the surface. These procedures were continued until ten sheets were stacked together. The construct was cultured at 37°C in an atmosphere of 5% CO_2_ and 95% relative humidity for 4 weeks. After that, the implantation was carried out. Figure 1 provides a brief summary of the construction process of implantation patch. The implantation patch was about 2 cm × 0.4 cm respectively in diameter and thickness. All constructions made by the same method in order to acquire similar sizes of implantation patches.

**Figure 2:**
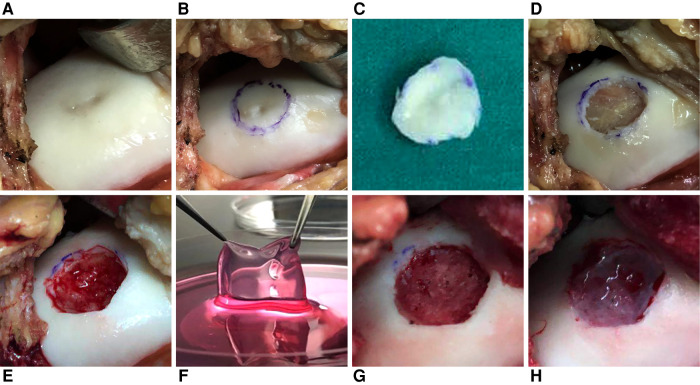
Mini-arthrotomy, curettage, and implantation in a patient (female; 36 years; left knee; BMI = 19.05 kg/m^2^; other surgery: bone grafting). (**A**) The cartilage defect was exposed. (**B,C**) The unhealthy cartilage was marked and removed. (**D**) The subchondral bone was exposed, and cystic degeneration could be seen. (**E**) The cystic degeneration in subchondral bone was removed, and blood effusion could be observed. (**F**) The patch was placed in 0.9% saline <5 min before transplantation. (**G**) A contralateral autogenous posterior iliac bone graft was made. (**H**) The patch was placed into the defect. Fibrin glue was applied to the surface to fix the patch to the defect without suturing.

The second step was composed of mini-arthrotomy, curettage, and implantation. During this step (Figure 2), the unhealthy cartilage and subchondral bone plate were cleaned carefully to ensure blood exudation was absent and to leave the base smooth. The patch was placed in physiologic (0.9%) saline <5 min before transplantation. It was trimmed carefully to fit the defect exactly before placed onto the defect. Then, fibrin glue was applied to the surface to fix the patch to the defect without suturing. If a subchondral cystic defect occurred, then debridement was done. A contralateral autogenous posterior iliac bone graft was made, and the bone graft was transplanted into the subchondral defect.

Patients accepted routine rehabilitation after arthroscopy. Postoperative rehabilitation after the second step was far more critical. Initially, the patients accepted fixation using a locking hinged knee brace, and the knee was placed in extension for 2–3 days. The purpose was to prevent the patch from dislodging and allow stable adhesion between the patch and subchondral plate. After that, active and passive motions were allowed. First, non-weightbearing quadricep-strengthening exercises on a bed were recommended to patients. Then, a device to ensure continuous passive motion was used for 90–135 min daily until discharge from the hospital. The range of motion (RoM) was from 0° to 30° initially, and the upper-limit RoM was increased 5° per day until 90° (∼2 weeks). Afterward, the full RoM was allowed if patients did not feel pain. Partial-weightbearing with a walking aid within 6 weeks was allowed, after which full-weightbearing was allowed.

The preoperative and postoperative knee function was evaluated by four subjective evaluation systems: International Knee Documentation Committee Subjective Knee Evaluation Form (IKDC-SKEF) (14); Lysholm Scale (15); Western Ontario and McMaster Universities Osteoarthritis Index (WOMAC) (16); Knee Injury and Osteoarthritis Outcome Score (KOOS) (17). These systems have been demonstrated to be effective and sensitive for evaluation of the repair of articular cartilage (18–24) and have been used widely for evaluation of outcomes after ACI (25, 26–33). MR examination of the operated knee joint was undertaken on a 3.0-T MR scanner (Ingenia 3.0-T CX, Philips Healthcare, Best, the Netherlands) using a sixteen-channel phased-array coil. The quality of repaired tissue was evaluated by Magnetic Resonance Observation of Cartilage Repair Tissue (MOCART). It is a classification system established by Marlovits et al. in 2004 to analyze repaired tissue (34). Studies have indicated a correlation between the clinical outcome and MOCART score (35, 36). Some researchers consider it to be a reliable way to evaluate repaired tissue (37, 38).

**Figure 3:**
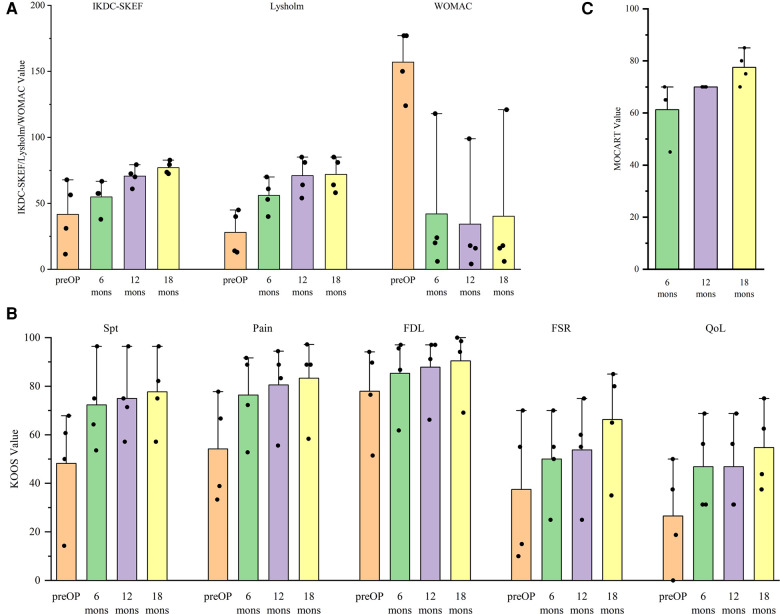
Variation in the trend of (**A**) international knee documentation committee subjective knee evaluation form (IKDC-SKEF), lysholm scale, western Ontario and mcMaster universities osteoarthritis Index (WOMAC), (**B**) knee injury and osteoarthritis outcome score (KOOS), and (**C**) magnetic resonance observation of cartilage repair tissue (MOCART) scores.

**Figure 4:**
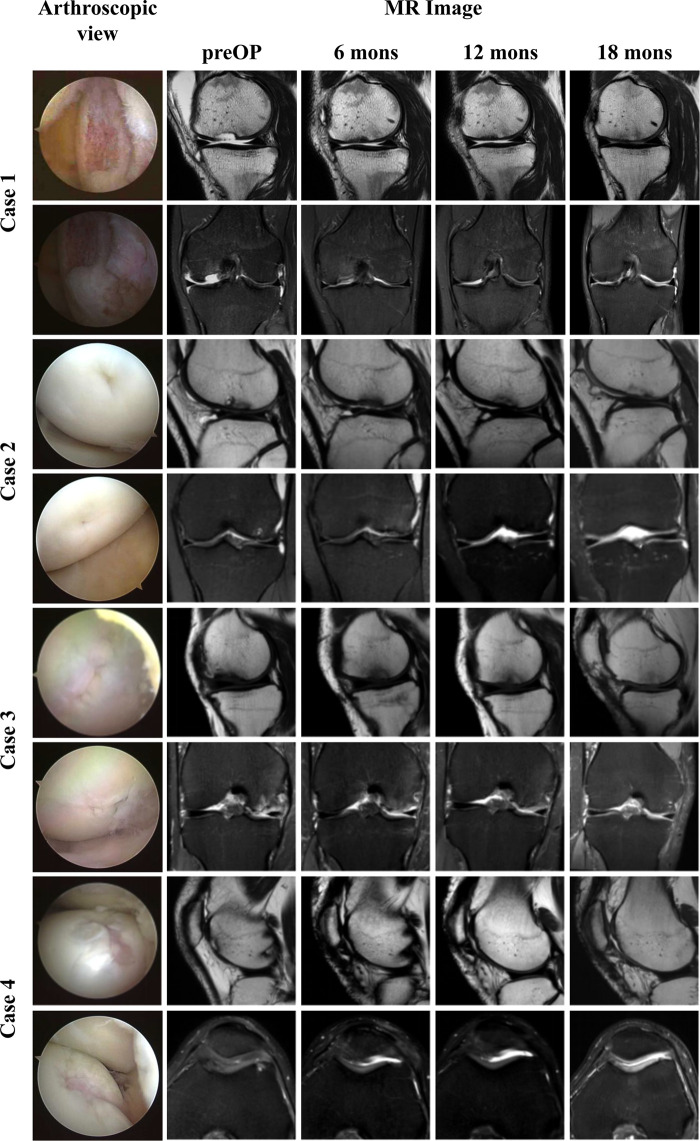
Arthroscopic view and MR images of four patients. The first column: arthroscopic view of cartilage defect. The second to fifth columns: Sagittal T2-weighted, coronal, or axial PD-weighted MR images of four patients from pre to 18 months after procedure.

Overall, the patients were all satisfied with the surgical treatments. All patients showed improvements on all clinical outcomes over the time (Figures 3A,B). As an exception, the scores of one patient were lower than those of other patients in IKDC-SKEF, Lysholm Scale, and KOOS. Accordingly, WOMAC scores were higher. His postoperative WOMAC score at 18-month was higher than that at 12-month, and was similar to the score at 6-month. He complained of knee joint pain after walking for a long time or recreational activities. The MOCART scores increased gradually after procedure (Figure 3C) for all patients. The MR images of patients are shown in Figure 4. As can be seen, the defect area decreased postoperatively, and the signal intensity of the repaired cartilage was close to that of healthy cartilage 12-month postoperatively. There was virtually no sclerosis of subchondral bone or edema 18-month postoperatively.

## Discussion

The improvements in IKDC-SKEF, Lysholm Scale, WOMAC, and KOOS were demonstrated postoperatively in all patients. The patients expressed satisfaction with the functional postoperative recovery after autologous chondrocyte patch implantation.

As seen from Figure 3B, the function score in sport and recreation (FSR) and quality of life (QoL) were lower than other parts in KOOS at all times. This means that the time needed for patients to return to sports and recreational activities after the procedure was longer than that for returning to activities of daily living. It still needs a long time to return to a higher QoL level. Pestka et al. and Erdle et al. showed that returning to low-intensity and moderate-intensity activities was feasible, but returning to high-intensity or identical-intensity activities to that before surgery was infeasible in 1 year (39, 40). Ebert et al. and Erggelet et al. advised their patients to return to contact and competitive activities 12-month postoperatively (41, 42). Furthermore, Niethammer et al. compared the rehabilitation process postoperatively. They found that patients who returned to sporting activities 12-month postoperatively showed significantly better clinical outcomes than those who returned to sporting activities before 12-month postoperatively (43). However, Kreuz et al. concluded that moderate-intensity sporting activities are an essential component of rehabilitation but should be undertaken at least 2–3 years after the surgical procedure (44). All of the above studies demonstrated that patients should not return to high-intensity activities too earlier. It may help surgeons and patients determine the appropriate time for returning to sporting activities postoperatively.

The MOCART scores increased gradually after the surgical procedure. These data are in accordance with the trend reported by Zak et al. and Niemeyer and colleagues (45, 46). It illustrated that the repaired cartilage tissue progressed towards a healthy morphology. MR examination is a non-invasive way to evaluate articular cartilage and has a sensitivity of ≤96% (47). MR features can show cartilage status directly. The MOCART score is a quantitative indicator to evaluate cartilage. However, Siebold et al. pointed out that a low MOCART score did not denote poor clinical results but, instead, we should not evaluate cartilage status using the MOCART score only (4).

In the present study, the mean total cost of procedure was 57693.2 (range, 55905.2–61644.2) CNY. This figure included the fees for preoperative consultation and preparation, surgical items (anesthesia, surgical supplies, duration of use of the operating room, drugs, and hospital fees), cell processing, and implantation patch fabrication. The costs of this procedure are significantly higher than those of knee arthroscopy (chondroplasty and debridement), microfracture (MFx), osteochondral transplantation (OCT), or osteochondral allograft (OCA) (6, 25, 48). However, arthroscopic chondroplasty and debridement cannot fundamentally tackle the problem. MFx leads to the synthesis of fibrocartilage instead of hyaline cartilage. The biomechanical property of the fibrocartilage is inferior to that of hyaline cartilage, which leads to a worse long-term outcome (4, 8, 9, 49–54). Donor-area complications (e.g., pain, discomfort, and formation of secondary bone defects) cannot be ignored after OCT (55).

Although ACI can lead to the synthesis of hyaline cartilage, the different generation of ACI have different advantages and disadvantages. Samuelson et al. demonstrated that ACI-P and ACI-C are cost-effective, and that the latter is marginally more cost-effective than the former (56). Their results are similar to those of Schrock and colleagues (25). Everhart et al. pointed out that the cost of MACI is similar or slightly higher than that of ACI-C (48). Several studies have pointed out that MACI can lead to satisfactory and reliable clinical outcomes (7, 9, 26, 27, 30, 57). It has other advantages: homogeneous distribution; biocompatibility; appropriateness for large cartilage defects; relatively simple production process; easy to model; can be produced in differently sized and shaped membranes; straightforward surgical procedure; use of fibrin glue instead of suturing (6). Most importantly, it has been approved by the Chinese government. According to the three generations of ACI, only ACI-P and MACI have been approved in China. The patch in this study was easy to prepare and not technically demanding. Due to the disadvantages of ACI-P (e.g., complex surgical procedure, highly invasive, inhomogeneous distribution, weak biomechanical property), we believe that our procedure is an excellent way to treat patients with a focal articular-cartilage defect in knee joint.

Interestingly, the changes of WOMAC score of one patient were different from those of other patients. He complained of knee joint pain after walking for a long time or recreational activities from 12- to 18-month postoperatively. During a recent follow-up (18-month postoperatively), we undertook a comprehensive examination of the operated knee. We discovered that the location of the knee joint pain for this patient was not in accordance with the location of the cartilage defect. We found hyperosteogeny according to preoperative radiography (Supplementary Figure S1). The hyperosteogeny and appearance of osteoarthritis became more marked with time.

There are likely two explanations for this problem. First, the indications for the procedure should be stricter. Even though the patient was aged only 46 years, the appearance of osteoarthritis was present preoperatively. Although the clinical outcome was good, it did not proceed as expected. Niemeyer et al. considered that diffuse lesions (e.g., osteoarthritis) should not be included in the indication for ACI (54). Conversely, Minas and collaborators considered that patients with early-stage osteoarthritis can accept ACI (58). Rosenberger et al. pointed out that the failure rates of ACI in older and younger patient groups are similar if the cartilage defect is focal (59). Second, the progress of intrinsic osteoarthritis for this patient influenced the effect postoperatively. Osteoarthritis progression may be fast in some patients, even if the procedure has been done and the defect has been repaired. A focal cartilage defect is a risk factor for osteoarthritis development (54). The procedure can be a powerful tool for delaying osteoarthritis progression, but intrinsic osteoarthritis cannot be delayed (58). Therefore, patients aged >40 years with osteoarthritis may carry the risk of a poor clinical outcome after the procedure, and whether to have a procedure should be considered carefully.

## Conclusion

Patients undergoing autologous chondrocyte patch (fabricated *via* the sandwich technique) implantation demonstrated clinical improvement and good quality of repaired tissue postoperatively. The procedure is an efficient and cost-effective treatment for knee joint cartilage defect in this report. In addition, patients with osteoarthritis carry the risk of a poor outcome after the procedure, and whether to have a procedure should be considered carefully. Large studies with long-term follow-up are also needed.

## Data Availability

The raw data supporting the conclusions of this article will be made available by the authors, without undue reservation.
