# Supplementary material for: Four cases report: Treatment of knee joint cartilage defects using autologous chondrocyte patch implantation
Source: Front Surg. 2022 Nov 8;9:1015091. doi: 10.3389/fsurg.2022.1015091 (PMC9679023; doi:10.3389/fsurg.2022.1015091)
Supplement: Supplementary file 1 [file Table1.docx]

Supplementary Material

# Supplementary Figure

# Figure Legend

**Supplementary Figure 1.** Radiographic examination images of a patient (male; 47 years; right knee; BMI = 26.83 kg/m^2^; no other surgical procedures). (A, B) Preoperative radiograph: hyperosteogeny and cartilage defect can be seen. (C, D) Postoperative radiograph (18 months): hyperosteogeny and slight joint space narrowing can be seen. The defect has been repaired (white arrowhead) and is nearly the same as the adjacent joint.

# Supplementary Table

**Supplementary Table 1.** Demographic Characteristics of the Cases

| **Characteristic** | **Results** |
| --- | --- |
| Total number of patients, n | 4 |
| **Demographic** |  |
| Age, years |  |
| Mean (range) | 36.5 (26–47) |
| Median | 36.5 |
| Sex, n (%) |  |
| Male | 3 (75.0) |
| Female | 1 (25.0) |
| Knee involved, n (%) |  |
| Left | 3 (75.0) |
| Right | 1 (25.0) |
| BMI, kg/m2 |  |
| Mean (range) | 24.5 (19.1–29.9) |
| Median | 24.6 |
| Smokers, n (%) |  |
| Smokers | 1 (25.0) |
| Non-smokers | 3 (75.0) |
| Previous surgical procedures, n (%) |  |
| Yes | 1 (25.0) |
| No | 3 (75.0) |
| **Defects** |  |
| Number of defects, n (%) |  |
| One defect | 4 (100.0) |
| Two or more defects | 0 (0) |
| Defect size, cm2 |  |
| Mean (range) | 3.2 (1.0–6.3) |
| Median | 2.8 |
| Defect localization, n (%) |  |
| Medial femoral condyle (MFC) | 2 (50.0) |
| Lateral femoral condyle (LFC) | 2 (50.0) |
| **Treatment** |  |
| Etiology, n (%) |  |
| Trauma (≤1 year) | 1 (25.0) |
| Trauma (>1 year) | 2 (50.0) |
| Unknown | 1 (25.0) |
| ICRS grade | III–IV |
| Other surgical procedures, n (%) |  |
| No | 2 (50.0) |
| Bone grafting | 2 (50.0) |
| Time between collection and implantation, weeks |  |
| Mean (range) | 6.3 (4–8) |
| Median | 6.5 |
| Follow-up duration, months |  |
| Mean (range) | 25.3 (18–33) |
| Median | 25.0 |
| Satisfaction, scores |  |
| Knee joint |  |
| Mean (range) | 9 (7–10) |
| Median | 9.5 |
| Overall |  |
| Mean (range) | 9.8 (9–10) |
| Median | 10.0 |
| Total costs, CNY (Chinese Yuan) |  |
| Mean (range) | 57693.2 (55905.2–61644.2) |
| Median | 56611.7 |
