# Supplementary figures and images for: Four cases report: Treatment of knee joint cartilage defects using autologous chondrocyte patch implantation
Source: Front Surg. 2022 Nov 8;9:1015091. doi: 10.3389/fsurg.2022.1015091 (PMC9679023; doi:10.3389/fsurg.2022.1015091)

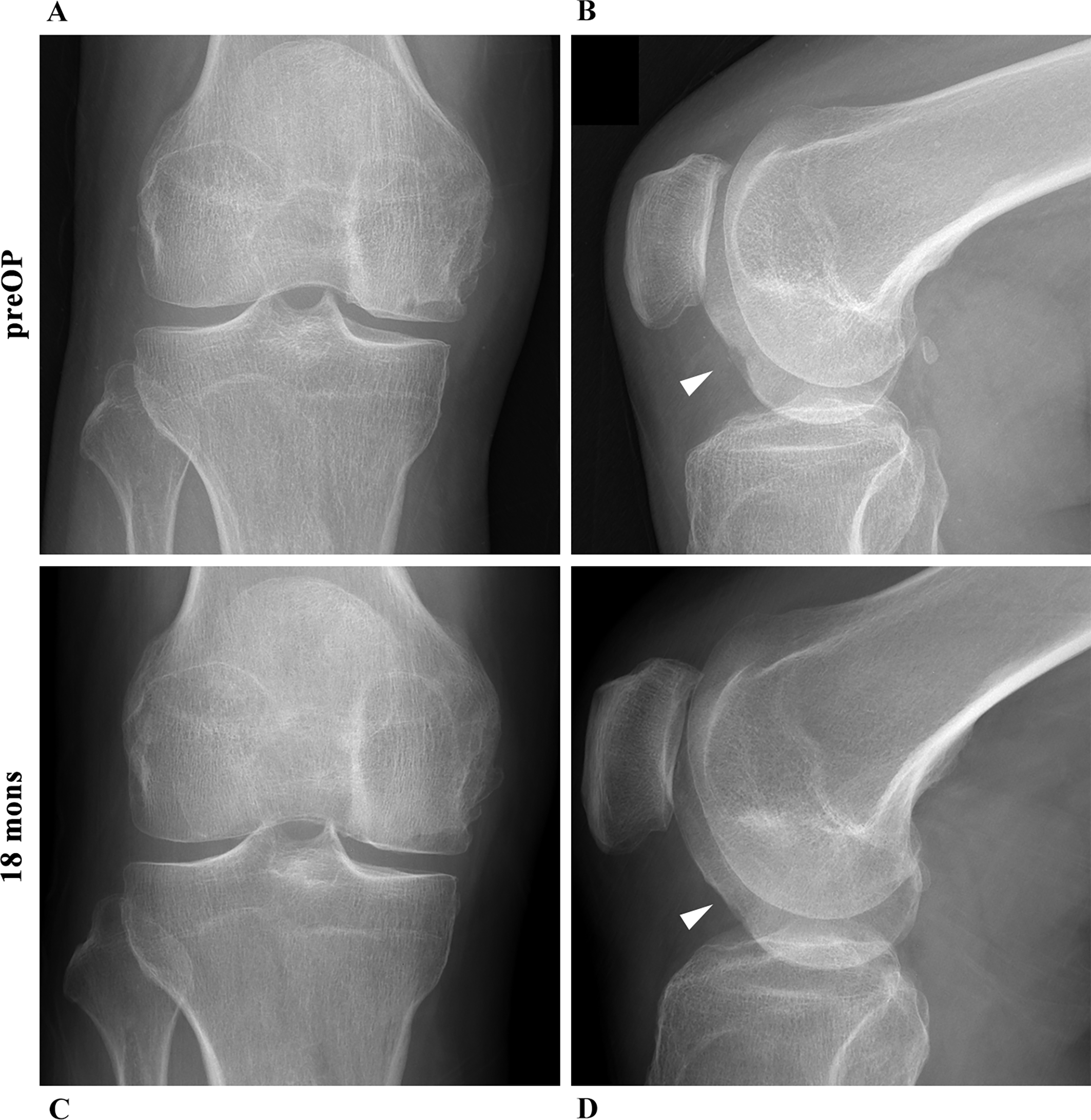

Supplement: Supplementary file 2 [file Image1.png]
